# Recent and projected precipitation and temperature changes in the Grand Canyon area with implications for groundwater resources

**DOI:** 10.1038/s41598-020-76743-6

**Published:** 2020-11-12

**Authors:** Fred D Tillman, Subhrendu Gangopadhyay, Tom Pruitt

**Affiliations:** 1 U.S. Geological Survey, Arizona Water Science Center, Tucson, AZ USA; 2 Reclamation, Water Resources Engineering and Management Group, Denver, CO USA

**Keywords:** Climate sciences, Hydrology

## Abstract

Groundwater is a critical resource in the Grand Canyon region, supplying nearly all water needs for residents and millions of visitors. Additionally, groundwater discharging at hundreds of spring locations in and near Grand Canyon supports important ecosystems in this mostly arid environment. The security of groundwater supplies is of critical importance for both people and ecosystems in the region and the potential for changes to groundwater systems from projected climate change is a cause for concern. In this study, we analyze recent historical and projected precipitation and temperature data for the Grand Canyon region. Projected climate scenarios are then used in Soil Water Balance groundwater infiltration simulations to understand the state-of-the-science on projected changes to groundwater resources in the area. Historical climate data from 1896 through 2019 indicate multi-decadal cyclical patterns in both precipitation and temperature for most of the time period. Since the 1970s, however, a significant rising trend in temperature is observed in the area. All 10-year periods since 1993 are characterized by both below average precipitation and above average temperature. Downscaled and bias-corrected precipitation and temperature output from 97 CMIP5 global climate models for the water-year 2020–2099 time period indicate projected precipitation patterns similar to recent historical (water-year 1951–2015) data. Projected temperature for the Grand Canyon area, however, is expected to rise by as much as 3.4 °C by the end of the century, relative to the recent historical average. Integrating the effects of projected precipitation and temperature changes on groundwater infiltration, simulation results indicate that > 76% of future decades will experience average potential groundwater infiltration less than that of the recent historical period.

## Introduction

**Figure 1:**
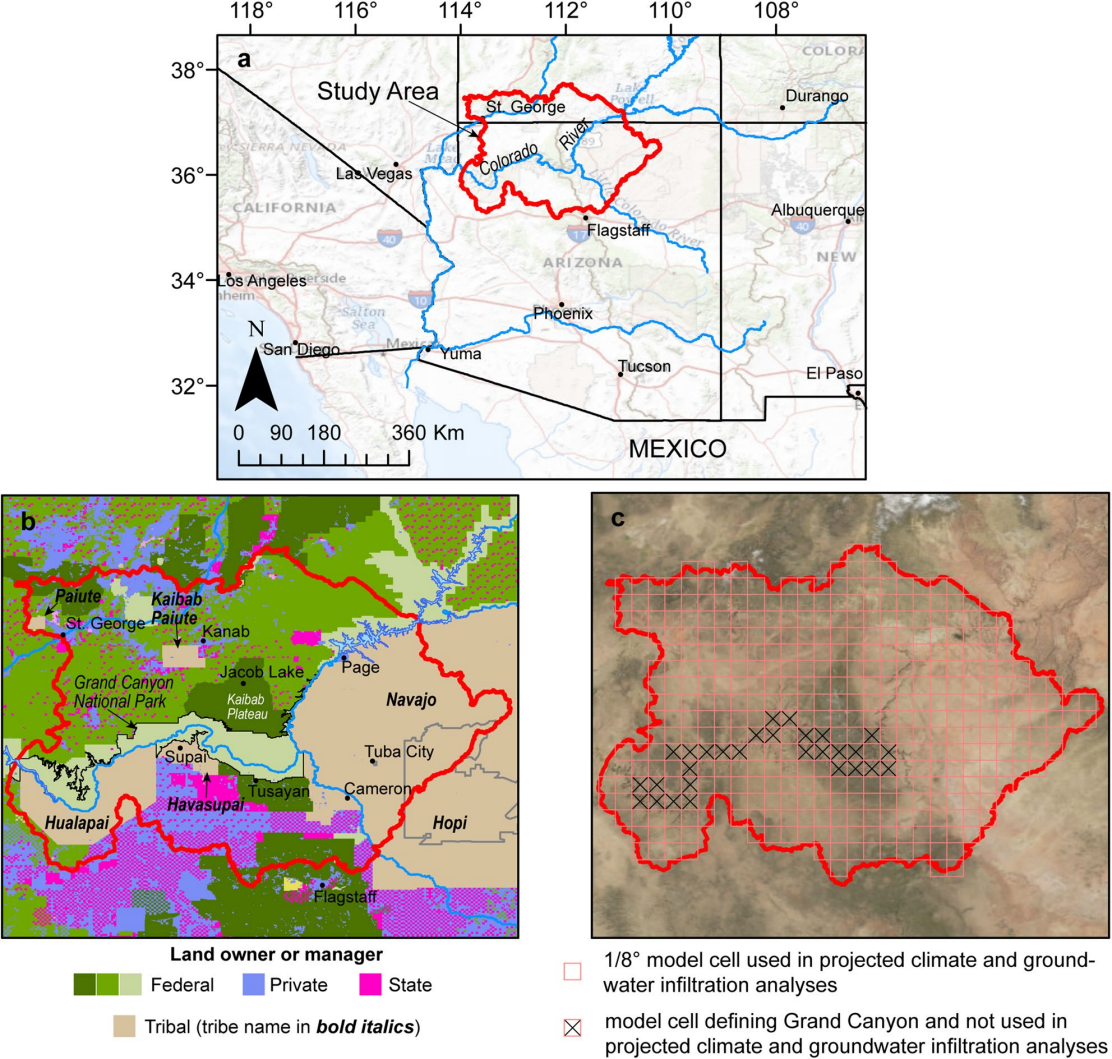
Grand Canyon study area within the southwestern U.S. **(a)**, land ownership or management within the study area^3,4^ **(b)**, and projected climate and groundwater infiltration model cell size **(c).** Maps created in Esri ArcMap 10.6.1. Basemap images in **(a,c)** from public domain USGS National Map.

The Grand Canyon in northern Arizona was formed by tectonic uplift and subsequent erosion by the Colorado River, which supplies the industrial, agricultural, and domestic water needs of more than 35 million people in the western U.S. and Mexico^1^. Most communities in the immediate Grand Canyon area, however, do not have rights or access to water from the fully apportioned river and thus are dependent upon groundwater to meet all water needs. Havasu Springs near Supai Village (Fig. 1) discharges about 1.7 m^3^/s from the Redwall-Muav aquifer into the world famous blue-green waters of Havasu Creek. Supai Village, home of the Havasupai Tribe, is entirely dependent upon groundwater issuing from Havasu Springs for irrigation and domestic use. Grand Canyon National Park is visited by over 6 million visitors each year^2^ and is also entirely dependent upon groundwater from springs. Other communities in the Grand Canyon region that depend entirely upon groundwater for their water needs include the Hualapai Nation and the towns of Tusayan, Williams, and Jacob Lake (Fig. 1). The security of future groundwater supplies is of critical importance to these communities.

Observed precipitation and temperature trends over the latter part of the twentieth century indicate that the southwestern United States (U.S.) is moving towards a drier, warmer state^5–10^. Observational data and modeling results suggest that the North American monsoon (NAM), which accounts for 30–50% of annual precipitation in the southwestern U.S., is becoming more extreme in intensity^5^. Transient inverted troughs, which trigger severe weather during the NAM in the southwestern U.S., have increased in density during late NAM season in the region over the last 60 years, although these troughs may have not been as important in initiating and organizing monsoon convection during recent warm seasons^7^. Projected future climate in the western U.S. is expected to be characterized by increased warming trends and increased drought severity^6,11,12^, although these projected trends vary somewhat in the region, notably by latitude^13^. Studies of the potential effects of projected climate change on groundwater resources in the western U.S. generally conclude that a range of effects are expected, with wet areas expected to get wetter and dry areas to become drier. The southern portions of the western U.S. are expected to receive less groundwater recharge and the northern portions may receive more^13,14^. Conclusions from large, regional-scale summaries of projected climate information and potential effects on groundwater resources, however, may mask important differences in smaller sub-regions within their study areas. For example, Tillman et al.^15^ found that projected increases in groundwater infiltration, relative to recent historical averages, in the upper Colorado River basin in the coming century were not shared equally within the basin. Sub-regions within the basin, particularly the northernmost areas, are projected to have somewhat greater groundwater infiltration than the basin average, while other sub-regions, particularly in the southernmost areas, are projected to experience a substantial reduction in groundwater infiltration in future climates^16^. For this reason, focus-area studies of particularly groundwater-dependent sub-regions, such as the Grand Canyon area, are important.

The objectives of this Grand Canyon area study are to investigate available historical climate data to understand recent temperature and precipitation trends and variability, to investigate global climate model projected climate data to understand expected climate change for the area, and to incorporate projected climate data into a groundwater infiltration model to understand the potential for climate impacts on groundwater resources in the area.

### Study area

The groundwater system in the Grand Canyon area is complex and poorly understood. Relatively shallow perched groundwater in the Permian-age Coconino Sandstone at a depth of about 300 m is found discontinuously in many parts of the region, but in varying quantities. A deeper, more productive regional groundwater system, referred to as the Redwall-Muav aquifer, is in the Mississippian-age Redwall Limestone, the Devonian-age Temple Butte Formation, and the Cambrian-age Muav Formation > 1000 m below land surface. The Redwall-Muav aquifer is present throughout the region except where the units are cut by the Grand Canyon itself. Structural features may separate the groundwater system into mostly independent subsystems^17^. The timing of groundwater flow from areas of recharge to discharge locations varies greatly across the region, ranging from days to months for some springs along the Kaibab Plateau^18,19^ to thousands of years at other springs and wells in the area^20,21^. Owing to the remoteness of the area and the depth to groundwater, few wells are available with which to evaluate groundwater flow paths and basin boundaries. For this study, topographic watersheds were used as a proxy for the extent of the underlying groundwater system in the Grand Canyon area (Fig. 1a). U.S. Geological Survey (USGS) 10-digit Hydrologic Unit Code drainages (HUC10s) that drain to the Colorado River between the Grand Wash Cliffs in the west (near the eastern edge of Lake Mead) and Lees Ferry in the east were combined to create the study-area boundary for this investigation.

The study area is located on the Colorado Plateau in northern Arizona and southern Utah (Fig. 1a). Land within the study area is owned or managed by the Federal government (50%), Tribal Nations (32%), private interests (11%), and state agencies (7%). The study area encompasses all of the Havasupai, Kaibab Paiute, and Paiute Reservations, 94% of the Hualapai Reservation, 28% of the Hopi Reservation, and 20% of the Navajo Reservation (Fig. 1b). Land surface elevations are varied in the area, with broad, flat plateau surfaces at ~ 1500 m elevation above sea level, canyon floors from ~ 400–900 m elevation from west to east, and the Kaibab Plateau rising to over 2700 m. The climate of the study area varies with elevation, with warmer and drier lower elevation areas and cooler and wetter higher elevation areas. Accordingly, excess precipitation available for groundwater recharge is primarily found in higher elevations of the study area including the north slopes of the San Francisco Mountains near Flagstaff, Arizona and on the Kaibab Plateau north of Grand Canyon^22–24^.

### Data and methods

Historical precipitation and temperature for the study area were evaluated using the gridded 5 km Global Historical Climatology Network (GHCN)-Daily Temperature and Precipitation Dataset (nClimGrid)^25,26^. The nClimGrid dataset uses station data from the Global Historical Climatology Network with temperature bias correction and climatologically aided interpolation to address topographic and network variability, resulting in a dataset appropriate for evaluating regional climate trends^25,26^. Precipitation data in the nClimGrid dataset are modeled as a function of locational variables of latitude, longitude, and elevation^26^. Monthly gridded climate data, developed from the daily dataset, at the ~ 5 km spatial scale were available from 1896 through 2019. Historical climate data for the study area were aggregated into water year (October–September), 5-year moving average, and 10-year moving average periods for analysis.

Simulated future precipitation and temperature data for the study area were available through the year 2099 for 97 climate projections from the Coupled Model Intercomparison Project phase 5 (CMIP5) multi-model archive (Supplementary Table S1). Each of the 97 ensemble members are results from a global climate model (GCM) run using one of four future emission scenarios known as a Representative Concentration Pathway (RCP). The four emission scenarios are for radiative forcing levels of 8.5, 6, 4.5, and 2.6 W/m^2^ by the end of the century^27^. RCP8.5 represents a high baseline emission scenario that presumes no climate policy is agreed upon in the coming century and “business as usual” conditions prevail. RCP2.6 envisions a fairly rapid decrease in carbon emissions in the early part of this century with a subsequent stabilization of atmospheric CO_2_ concentrations by mid-century. Two medium stabilization scenarios (RCP4.5 and RCP6) project future emissions between these end members^27^. In this investigation, the range of future emission scenarios are all considered equally likely. GCMs are typically run at coarse spatial resolutions of ~ 100–200 km so there is a need to downscale GCM climate output to finer spatial scales for climate impact assessments. There is a continuum of downscaling methods ranging from statistical approaches to physically based modeling and the respective approaches are broadly referred to as statistical and dynamical downscaling methods. This study uses the statistical downscaling Bias-Correction and Spatial Disaggregation method^28^, referred to as BCSD, to develop monthly precipitation and temperature fields at 1/8° × 1/8° (latitude × longitude) spatial resolution from the GCM native scales to be consistent with earlier analyses conducted for the upper Colorado River basin^15,16^, though other statistically downscaled CMIP5 projections (e.g., using the Localized Constructed Analog, LOCA)^29,30^ are also currently available. Little difference is observed in the study area between downscaled climate data using the BCSD and LOCA methods^31^. Although widely used in hydrologic investigations, statistically downscaled climate data were shown to underestimate the mean and standard deviation of precipitation and overestimate the number of daily events as compared with a dynamically downscaling method in a limited study using three GCMs in the general vicinity of the current study area^32^. Convection permitting model (CPM) simulations also have been used to investigate projected changes in precipitation in the southwestern United States^9^. CPMs can capture observed changes in precipitation extremes, such as changes in North American monsoon precipitation intensity^9^, and explicitly resolve physical processes like convective organization and orographic precipitation^8,10^.

**Figure 2:**
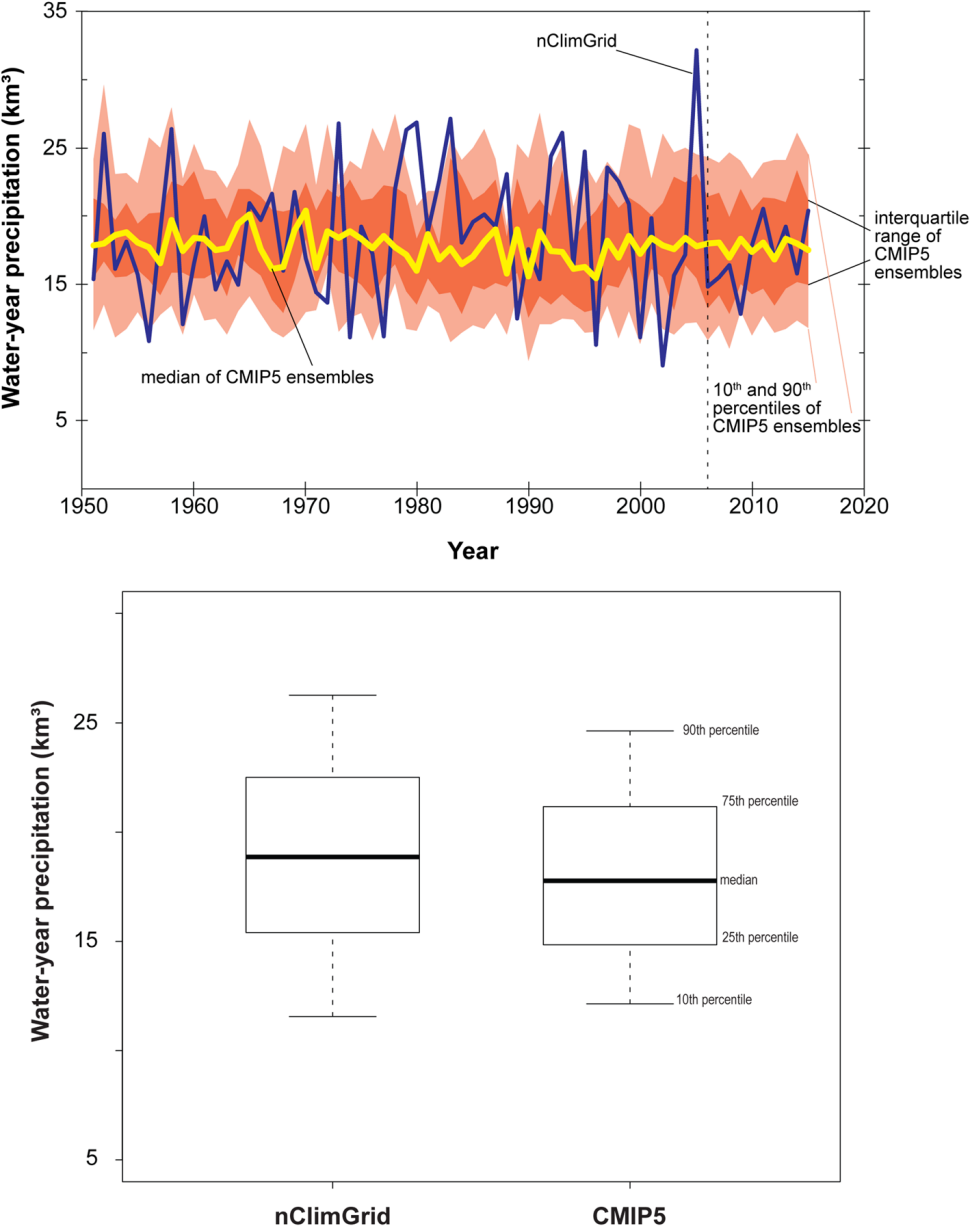
Comparison of distribution of global climate models (CMIP5) historical precipitation to gridded Global Historical Climatology Network (nClimGrid)^25,26^ precipitation for the Grand Canyon study area including time series comparison (top panel) and comparison of distributions of all water years (bottom panel). Vertical line at year 2005 in top panel indicates end of bias correction of GCM data.

While the scale of climate models does not allow GCMs to capture orographic effects in detail, statistical downscaling techniques that train-on and bias-correct-to observational data, which are “orographically aware”^31^, allow for downscaled GCM precipitation and temperature to account for orographic effects. CMIP5 historical precipitation downscaled using BCSD is consistent with nClimGrid historical precipitation for the Grand Canyon study area and the distributions of water-year precipitation between the downscaled GCMs and observed data are not statistically different over their common historical period (Wilcoxon rank sum, p-value = 0.33; Fig. 2). Comparing ten-year moving averages of historical precipitation, nClimGrid observed and median downscaled GCM results are within − 9.7% to 22.3% of each other over the 46 ten-year periods in common (Supplementary Fig. S1).

A historical resampling and scaling technique^33^ was subsequently used to disaggregate the monthly precipitation and temperature data to daily values. Projected daily precipitation and temperature data are available from the downscaled climate and hydrology projections archive hosted by Lawrence Livermore National Laboratory^34^ (https://gdo-dcp.ucllnl.org/downscaled_cmip_projections/dcpInterface.html).

While evaluating the current understanding of projected climate for the Grand Canyon area is by itself a useful endeavor, integrating the combined effects of projected temperature and precipitation is required to understand possible future changes in groundwater infiltration in the area. To investigate the potential for projected climate effects on groundwater resources in the study area, projected climate data from the 97 CMIP5 ensemble members were used in the Soil–Water Balance (SWB) groundwater infiltration model^35^. The SWB model estimates potential groundwater infiltration by calculating water balance components using a modified version of the Thornthwaite-Mather^36,37^ soil–water-balance approach (see Supplementary Text S1 for model details and limitations). Groundwater infiltration is calculated on a daily time step as the difference between sources and sinks of water and change in soil moisture. Sources of water in the SWB model include rainfall, snowmelt, and inflow from other model cells while sinks of water include interception, outflow to other model cells, and evapotranspiration (ET). The SWB groundwater infiltration model has been used in several regional groundwater studies in the U.S. including the High Plains Aquifer^38^, the Lake Michigan Basin^39^, basins in Wisconsin^40^ and Minnesota^41^, the Northern Atlantic Coastal Plain aquifer system^42^, and the upper Colorado River basin^15,16^.

Owing to the absence of aquifer units within the Grand Canyon, projected climate data for the 97 CMIP5 ensemble members, and subsequent groundwater infiltration simulation results, were compiled and analyzed over a slightly smaller subset of the study area that does not include Grand Canyon itself (Fig. 1c). Projected climate data and simulated groundwater infiltration were aggregated into water years and then subsequently averaged over 10-year periods, moving every year. The 10-year moving average smooths out inter-annual variability and makes trends more apparent. Median values for the 97 climate ensemble members and resulting simulated groundwater infiltration are used to describe the central tendency of the climate and infiltration results, with the interquartile range (25th and 75th percentiles) of results presented to illustrate variability. Results for both the projected climate data and simulated groundwater infiltration are presented as a percent of the average of available CMIP5 historical data (water years 1951–2015). Comparing these results between future and past 10-year moving averages addresses the question “how might climate and groundwater infiltration in any future decade differ from conditions experienced in decades since 1951?”.

Descriptive, correlative, and hypothesis-testing statistics were performed using the R statistical code^43^. A significant statistical test result was defined as having a p-value < 0.05.

## Results and discussion

### Historical climate data

**Figure 3:**
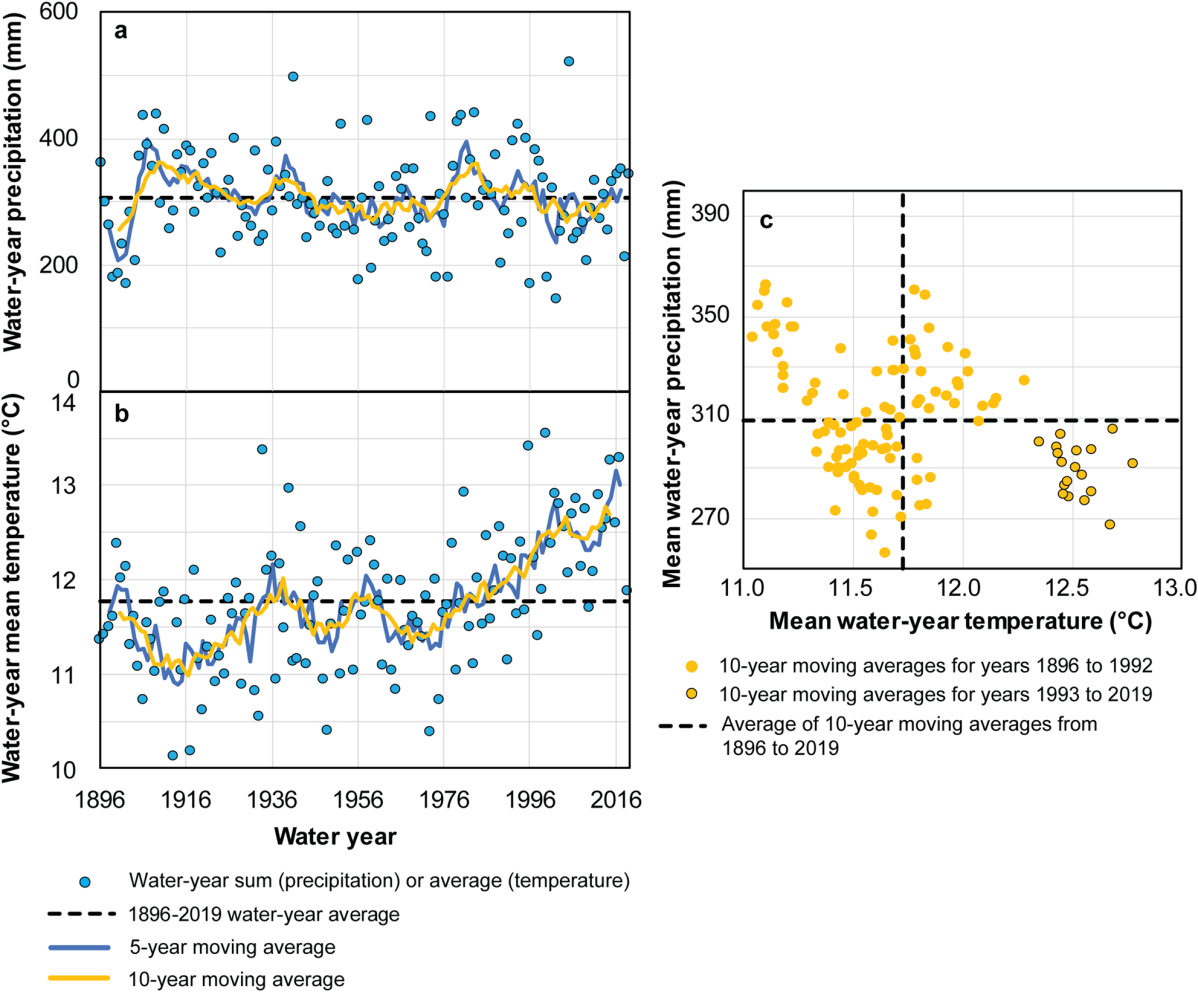
Historical precipitation **(a) **and temperature **(b)** data for the Grand Canyon study area, and relation between 10-year moving average of precipitation and temperature **(c)**. Moving averages are plotted in the middle year of averaging period.

Historical precipitation and temperature data for the study area were available from water year 1896 through 2019 (Fig. 3). Patterns are difficult to observe in individual water-year values, but 5- and 10-year moving averages illustrate a 10–20-year cycle in precipitation in the study area (Fig. 3a). This cyclical pattern has also been observed in other regions in the Western U.S.^44,45^ and is likely related to the Pacific quasi-decadal oscillation (QDO)^46^. The QDO describes cyclic, coupled evolutions of sea surface temperature and atmospheric circulation anomalies at 10–20-year time scales^47^. Ten-year moving averages of precipitation do not deviate substantially from the long-term (water year 1896–2019) average for the area, with a maximum wet period of 18% greater than average in the decade 1905–14 and a maximum dry period of 16% below average in the decade 1896–1905. More recently, beginning in water year 1993, 10-year moving averages of precipitation have been all below the long-term average.

Historical temperature data (Fig. 3b) also exhibit 10–20-year cyclical patterns in 5- and 10-year moving averages, at least until the latter quarter of the twentieth century. The maximum rise in 10-year moving average temperature cycles over most of the available historical data is 1.0 °C between decades 1912–21 and 1934–43. Beginning in water year 1971, a significant warming trend in 10-year moving averages of temperature in the study area is observed (Kendall’s tau = 0.83, p < 2.2 × 10^–16^), with a maximum rise to date of 1.3 °C. This warming trend in the Grand Canyon area has also been documented in other studies of the southwestern U.S.^6^. All other conditions remaining constant, increasing temperature would serve to increase evaporative demand and reduce soil moisture, leading to reduced water available for groundwater infiltration and replenishment of aquifers^11^. Since 1993, however, 10-year moving averages of temperature have been warmer than the historical average *and* precipitation has been drier than the historical average (Fig. 3c). The combined effects of both warmer and drier conditions observed recently in the study area would result in even less available water for groundwater recharge.

### Projected climate data

Projected precipitation and temperature results from 97 CMIP5 climate ensemble members were compiled and analyzed over a slightly smaller subset of the study area that does not include Grand Canyon itself (Fig. 1c). The median of the 10-year moving averages of all climate projections are evaluated and presented, with the interquartile range provided to illustrate variance among the climate models. Projected climate data are presented as a percentage of the water year 1951–2015 historical average.

**Figure 4:**
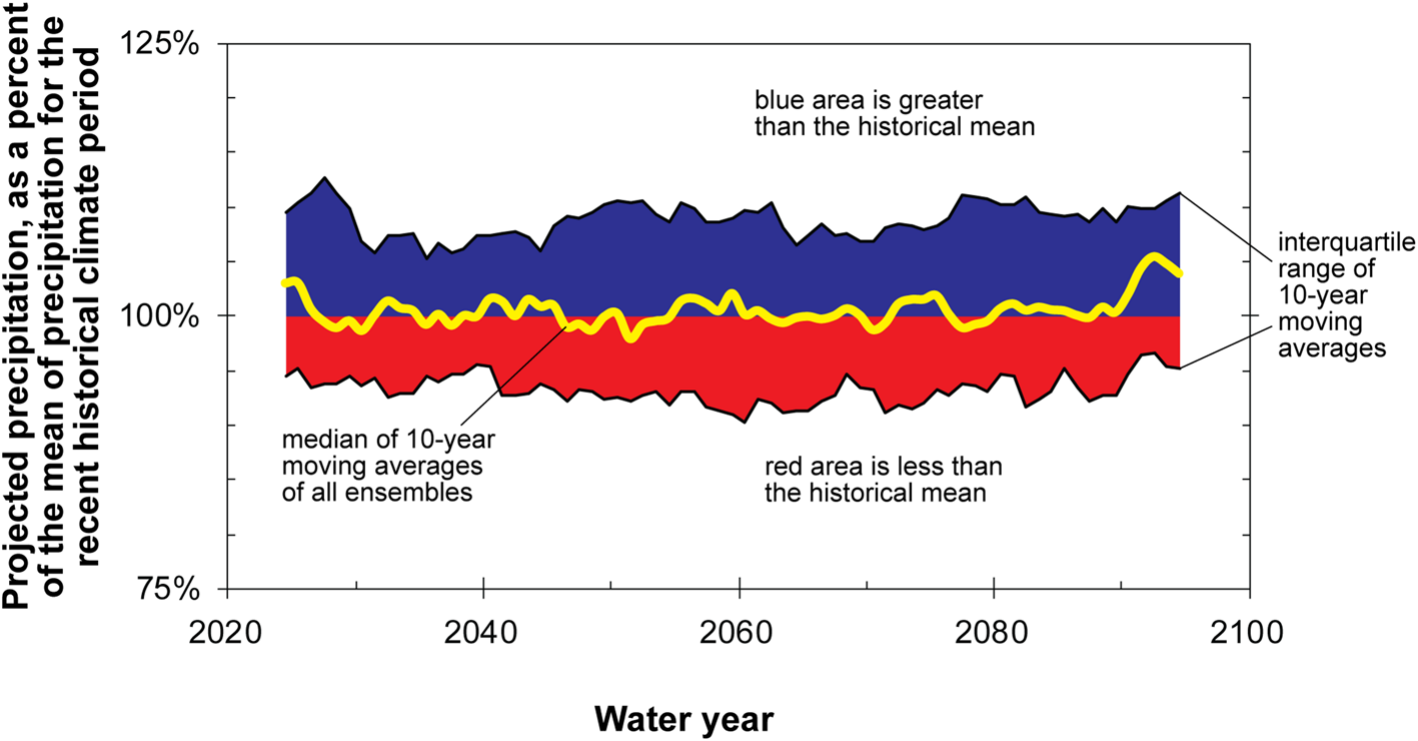
Projected precipitation data, relative to the 1951–2015 historical mean, for the Grand Canyon study area from 97 CMIP5 climate ensemble members investigated for this study. Medians of 10-year moving averages are plotted in the middle year of the averaging period.

Projected precipitation data from the climate ensemble indicate similar future precipitation patterns as in the recent historical period for the area (Fig. 4). Periods of below and above average precipitation are evident in the projected data, with more future 10-year periods that are wetter (68%) than the recent average than drier (32%). Deviations from the recent historical average are small, however, with a maximum decrease of 2% and a maximum increase of 5% during any 10-year period through water year 2099. Overall, projected precipitation for the Grand Canyon area is expected to be similar to the recent historical period, or slightly wetter during some decades, with an average over the water year 2020–2099 period of 100.6% of the water year 1951–2015 average.

**Figure 5:**
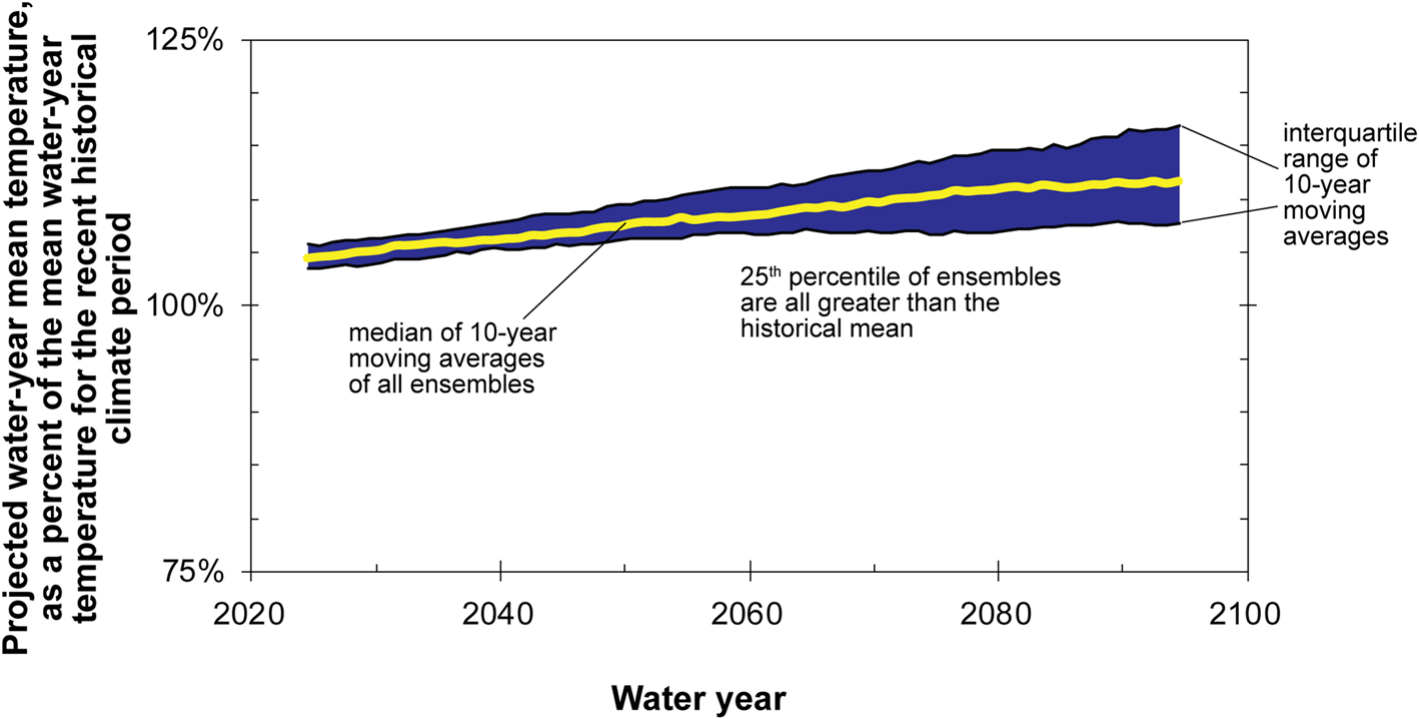
Projected mean temperature data, relative to the water-year 1951–2015 historical mean, for the Grand Canyon study area from 97 CMIP5 climate ensemble members investigated for this study. Medians of 10-year moving averages are plotted in the middle year of the averaging period.

Projected temperature data from the climate ensemble demonstrate a significant monotonic warming trend for the study area (Kendall’s tau = 0.98, p < 2.2 × 10^–16^; Fig. 5). By the end of the century, the 10-year moving average of annual average temperature for the study area is projected to be 12% (3.4 °C) warmer than the water-year 1951–2015 recent historical mean. This projected warming is in line with other studies of the Southwest U.S.^6^. Not only is the median of GCM climate projections warmer than the historical mean for all decades through the end of the century, but the 25th percentile (Fig. 5) and even the 1st percentile (not shown in Fig. 5) of climate projections also are uniformly warmer than the historical mean. With warmer temperatures projected across the Grand Canyon study area, evaporation and transpiration demands would be expected to increase, resulting in reduced groundwater infiltration for any given amount of precipitation, whether or not the amount of precipitation is expected to change in the area^12^.

### Projected groundwater infiltration

Projected precipitation and temperature data from the 97 CMIP5 climate ensemble members were used as input in SWB groundwater infiltration simulations to evaluate the potential for change in future groundwater systems in the Grand Canyon area in response to climate change. As previously described, the groundwater system in the Grand Canyon area is poorly understood and at present is the subject of ongoing investigations. There are, therefore, few resources with which to compare SWB groundwater infiltration results from this study. Reitz et al.^23^ present groundwater recharge estimates for the contiguous U.S. for the 2000–2013 time period. Average simulated groundwater infiltration from the current study for the CMIP5 climate ensemble over the same time period for the Grand Canyon study area (minus the inner gorge) is 0.684 km^3^, which is comparable with the Reitz et al.^23^ results of 0.672 km^3^ for the same area.

**Figure 6:**
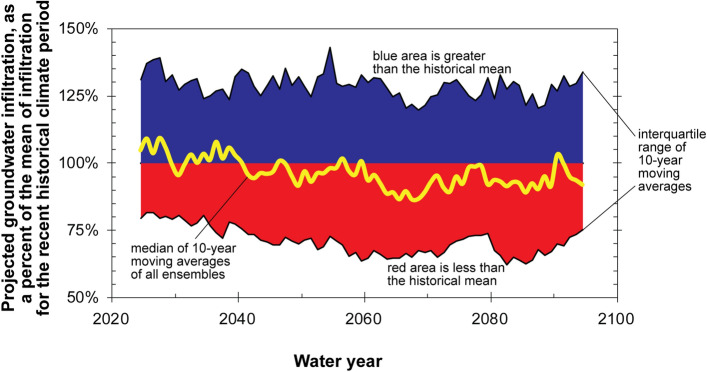
Simulated groundwater infiltration, relative to the water-year 1951–2015 historical mean, for the Grand Canyon study area using 97 CMIP5 climate ensemble members investigated for this study. Medians of 10-year moving averages are plotted in the middle year of the averaging period.

**Figure 7:**
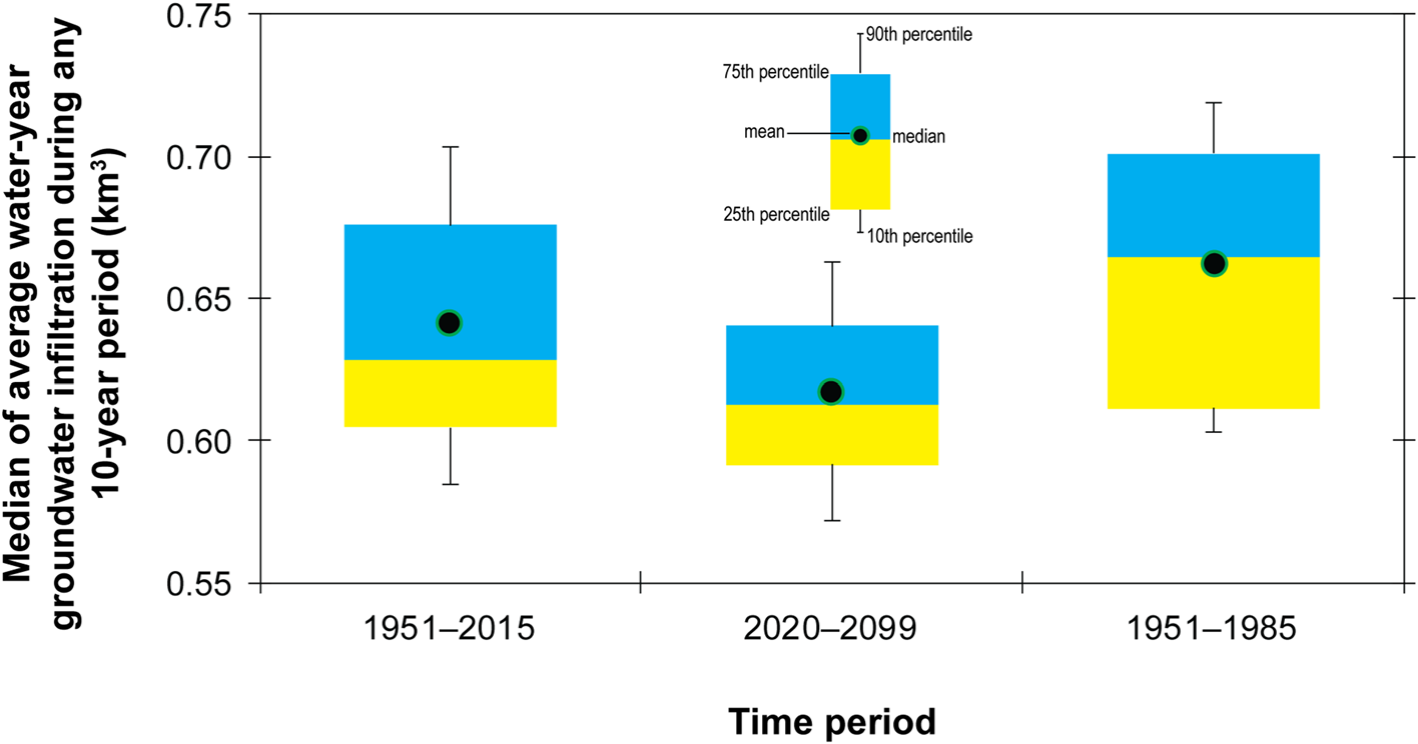
Distributions of medians of 10-year moving averages of simulated water-year groundwater infiltration in the Grand Canyon area for recent past (water-years 1951–2015 and 1951–1984) and future (water-year 2020–2099) time periods.

SWB simulated groundwater infiltration results using projected climate data indicate brief periods of above average infiltration in near-future time periods, followed by extended periods of below average infiltration throughout most of the remainder of the century (Fig. 6). Just over 76% of future 10-year periods are projected to have average potential groundwater infiltration less than that of the recent historical period (water years 1951–2015), with maximum declines as much as 14%. Comparing the distribution of simulated infiltration in all future decades with all 10-year periods in the water-year 1951–2015 recent historical period (Fig. 7), future groundwater infiltration in the Grand Canyon area is expected to be significantly less than that of the recent past (Wilcoxon rank sum, p = 0.008).

**Figure 8:**
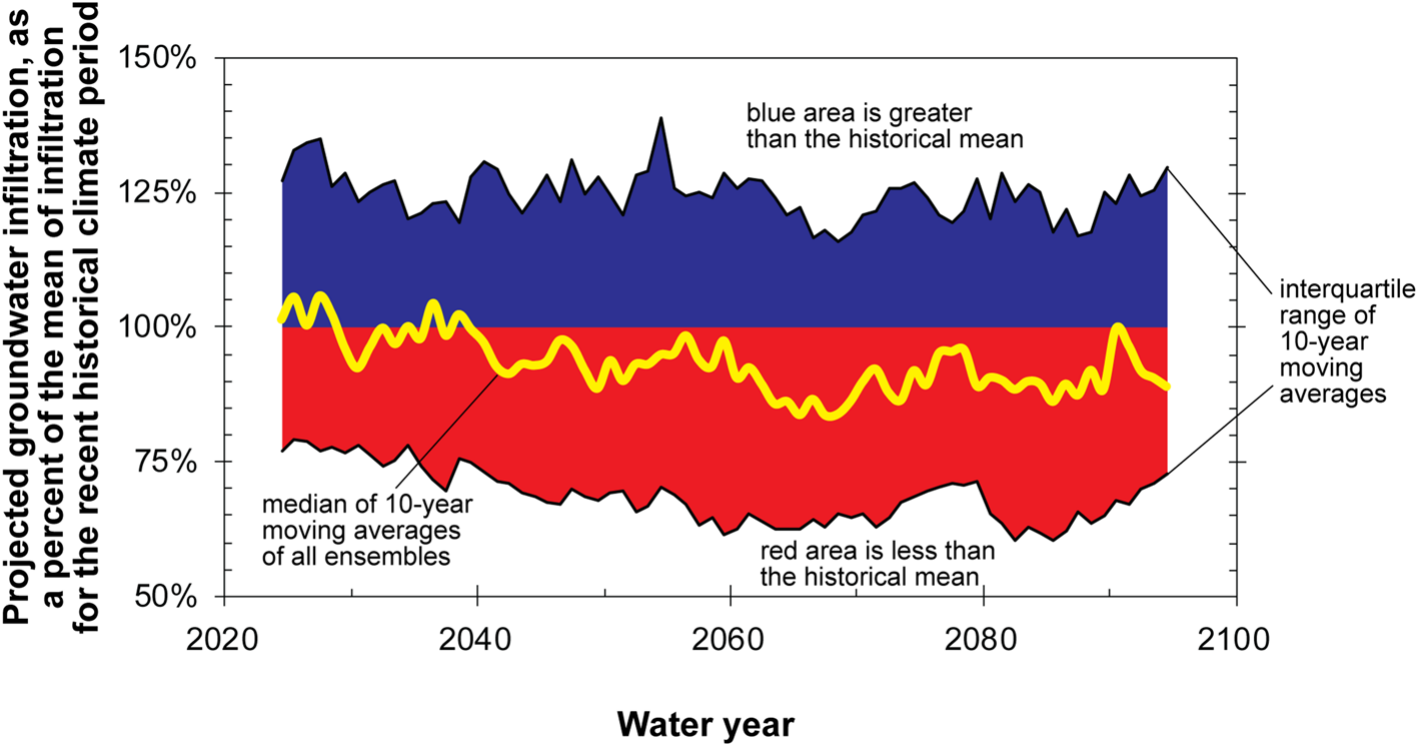
Simulated groundwater infiltration, relative to the water-year 1951–1984 historical mean, for the Grand Canyon study area using 97 CMIP5 climate ensemble members investigated for this study. Medians of 10-year moving averages are plotted in the middle year of the averaging period.

As discussed previously, the 1951–2015 historical period to which projected groundwater infiltration is compared in Fig. 6 includes a recent significant warming trend in temperature (Fig. 3b). If 10-year moving averages after the 1976–1984 decade, when the warming trend in 10-year moving averages of temperature crosses the historical mean in Fig. 3b, are excluded from the historical mean, then 89% of future decades are projected to experience potential groundwater infiltration that is less than that of the historical 1951–1984 time period (Figs. 7 and 8).

## Summary and conclusions

The Grand Canyon region in northern Arizona and southern Utah is home to the Havasupai, Hualapai, Navajo, Hopi, Kaibab Paiute, and Paiute Tribes. Grand Canyon National Park is visited by more than 6 million tourists annually. With limited access or rights to surface water in the area, groundwater supplies nearly all water needs of residents and visitors in the area. Additionally, groundwater discharging at hundreds of spring locations in and near Grand Canyon supports critical ecosystems in this mostly arid environment. The security of future groundwater supplies is, therefore, of great importance to these communities and ecosystems. This study of the Grand Canyon area investigated available historical climate data to understand recent trends in temperature and precipitation, investigated global climate model projected climate data to understand expected climate change, and incorporated projected climate data into a groundwater infiltration model to understand the potential for climate impacts on groundwater resources in the area.

Cyclical patterns are observed in historical climate data for the Grand Canyon area over the water-year 1896–2019 time period, at least until the last quarter of the twentieth century. Beginning in water year 1971, a significant warming trend in 10-year moving averages of temperature is observed in the study area with a maximum rise to date of 1.3 °C. Since water year 1993, 10-year moving averages of temperature have been warmer, and precipitation has been drier, than the recent historical (water-year 1896–2019) average. These combined effects of both warmer and drier conditions observed recently would result in reduced water available for groundwater recharge. Projected precipitation data from the climate ensemble indicate similar future precipitation patterns and magnitude as in the recent historical period for the area. Projected temperature for the Grand Canyon area, however, is projected to warm significantly throughout the rest of the twenty-first century. By 2099, median 10-year moving averages of mean temperature are projected to be 3.4 °C warmer than the water-year 1951–2015 recent historical mean. Results from Soil Water Balance groundwater infiltration model simulations indicate that > 76% of decades through the end of the century are expected to receive below average groundwater infiltration, relative to the recent past.

Owing to the complex groundwater system and uncertain groundwater flow paths in the Grand Canyon region, it cannot be said with certainty when climate effects on groundwater infiltration would be observed at particular springs or wells in the study area. If recent observed trends in climate data continue and current projections of future climate data hold true, however, then increasing temperature combined with little change in precipitation will result in less water available for recharging aquifer systems in the area. This general conclusion based on basic hydrologic principles is reinforced by groundwater infiltration simulation results that integrate changes in both projected precipitation and temperature data. While the timing of effects cannot be stated with certainty, these projected effects would be expected to be observed eventually in groundwater systems in the area. These results indicate a need to develop a greater understanding of the region’s groundwater system and for increased attention to managing the sustainability of groundwater resources in the region for future generations.

## Supplementary information

Supplementary Information.

## Data Availability

SWB groundwater infiltration modeling results for the Grand Canyon area are available at the U.S. Geological Survey ScienceBase web site^48^.
