# Supplementary material for: Recent and projected precipitation and temperature changes in the Grand Canyon area with implications for groundwater resources
Source: Sci Rep. 2020 Nov 12;10:19740. doi: 10.1038/s41598-020-76743-6 (PMC7664996; doi:10.1038/s41598-020-76743-6)
Supplement: Supplementary file 1 — Supplementary Information. [file 41598_2020_76743_MOESM1_ESM.pdf]

SCIENTIFIC REPORTS
natureresearch

Supplementary Information for

Recent and projected precipitation and temperature changes in the Grand Canyon area with implications for groundwater resources

Fred D Tillman[1*], Subhrendu Gangopadhyay[2], Tom Pruitt[2]

[1]U.S. Geological Survey, Arizona Water Science Center, Tucson, Arizona, USA; email: ftillman@usgs.gov
[2]Reclamation, Water Resources Engineering and Management Group, Denver, Colorado, USA

*Corresponding author

Contents of this file

Supplementary Table S1: CMIP5 multi-model projected climate ensembles and institutions providing model output used in the Grand Canyon area climate and groundwater resources investigation.

Supplementary Figure S1: Comparison of distribution of Global Climate Models (CMIP5) historical ten-year moving average precipitation to gridded Global Historical Climatology Network (nClimGrid) ten-year moving average precipitation for the Grand Canyon study area including time series comparison (top panel) and comparison of distributions of all ten-year moving averages (bottom panel).

Supplementary Text S1: Computational details on the Soil-Water Balance (SWB) groundwater infiltration model.

Supplementary Table S1. CMIP5 multi-model projected climate ensembles and institutions providing model output used in the Grand Canyon area climate and groundwater resources investigation.

Modeling Center or Group[a]	Model Name	Representative Concentration Pathway			
		2.6	4.5	6.0	8.5
BCC	BCC-CSM 1.1	✓	✓	✓	✓
BCC	BCC-CSM 1.1(m)		✓		✓
CCCMA	CanESM2	✓	✓		✓
CMCC	CMCC-CM		✓		✓
CNRM-CERFACS	CNRM-CM5		✓		✓
CSIRO-BOM	Access 1.0		✓		✓
CSIRO-QCCCE	CSIRO-mk3.6.0	✓	✓	✓	✓
FIO	FIO-ESM	✓	✓	✓	✓
INM	INM-CM4		✓		✓
IPSL	IPSL-CM5A-MR	✓	✓	✓	✓
IPSL	IPSL-CM5B-LR		✓		✓
LASG-CESS	FGOALS-g2	✓	✓		✓
MIROC	MIROC5	✓	✓	✓	✓
MIROC(2)	MIROC-ESM	✓	✓	✓	✓
MIROC(2)	MIROC-ESM-CHEM	✓	✓	✓	✓
MOHC	HadGEM2-AO	✓	✓	✓	✓
MOHC	HadGEM2-CC		✓		✓
MOHC	HadGEM2-ES	✓	✓	✓	✓
MPI-M	MPI-ESM-LR	✓	✓		✓
MPI-M	MPI-ESM-MR	✓	✓		✓
MRI	MRI-CGCM3	✓	✓		✓
NASA GISS	GISS-E2-H-CC		✓		
NASA GISS	GISS-E2-R	✓	✓	✓	✓
NASA GISS	GISS-E2-R-CC		✓		
NCAR	CCSM4(RSMAS)	✓	✓	✓	✓
NCC	NorESM1-M	✓	✓	✓	✓
NOAA GFDL	GFDL-CM3	✓	✓	✓	✓
NOAA GFDL	GFDL-ESM2G	✓	✓	✓	✓
NOAA GFDL	GFDL-ESM2M	✓	✓	✓	✓
NSF-DOE-NCAR	CESM1(BGC)		✓		✓
NSF-DOE-NCAR	CESM1(CAM5)	✓	✓	✓	✓

[a]BCC = Beijing Climate Center, China Meteorological Administration; CCCMA = Canadian Centre for Climate Modelling and Analysis; CMCC = Centro Euro-Mediterraneo per I Cambiamenti Climatici; CNRM-CERFACS = Centre National de Recherches Météorologiques /Centre Européen de Recherche et Formation Avancée en Calcul Scientifique; CSIRO-BOM = Commonwealth Scientific and Industrial Research Organization (CSIRO) and Bureau of Meteorology (BOM), Australia; CSIRO-QCCCE = Commonwealth Scientific and Industrial Research Organization in collaboration with Queensland Climate Change Centre of Excellence; FIO = The First Institute of Oceanography, SOA, China; INM = Institute for Numerical Mathematics; IPSL = Institut Pierre-Simon Laplace; LASG-CESS = LASG, Institute of Atmospheric Physics, Chinese Academy of Sciences and CESS,Tsinghua University; MIROC = Atmosphere and Ocean Research Institute (The University of Tokyo), National Institute for Environmental Studies, and Japan Agency for Marine-Earth Science and Technology; MIROC(2) = Japan Agency for Marine-Earth Science and Technology, Atmosphere and Ocean Research Institute (The University of Tokyo), and National Institute for Environmental Studies; MOHC = Met Office Hadley Centre (additional HadGEM2-ES realizations contributed by Instituto Nacional de Pesquisas Espaciais); MPI-M = Max-Planck-Institut für Meteorologie (Max Planck Institute for Meteorology); MRI = Meteorological Research Institute; NASA GISS = NASA Goddard Institute for Space Studies; NCAR = National Center for Atmospheric Research; NCC = Norwegian Climate Centre; NOAA GFDL = NOAA Geophysical Fluid Dynamics Laboratory; NSF-DOE-NCAR = Community Earth System Model Contributors

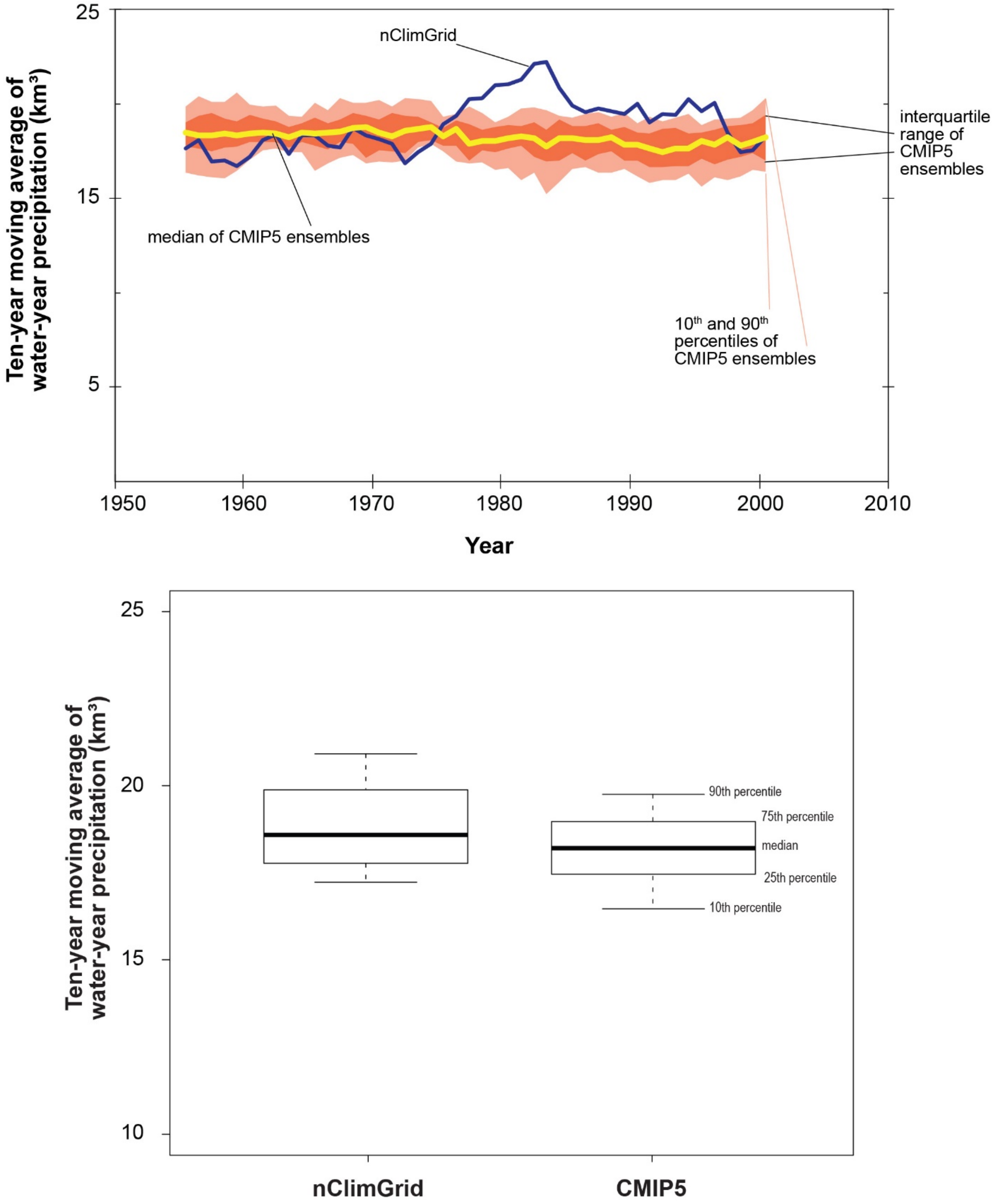

Supplementary Figure S1. Comparison of distribution of Global Climate Models (CMIP5) historical ten-year moving average precipitation to gridded Global Historical Climatology Network (nClimGrid) [1, 2] ten-year moving average precipitation for the Grand Canyon study area including time series comparison (top panel) and comparison of distributions of all ten-year moving averages (bottom panel).

Supplementary Text S1.

The Soil-Water-Balance (SWB) computer code [3] estimates spatial and temporal variations in groundwater recharge by calculating water balance components at daily time steps. SWB follows a modified Thornthwaite-Mather soil-water-balance accounting approach [4, 5] and recharge is estimated separately for each grid cell within the model domain. Sources and sinks of water within each grid cell are estimated based on climate data and landscape characteristics, and recharge is then estimated as the difference between the change in soil moisture and these sources and sinks:

$$\underbrace{(\text{rainfall} + \text{snowmelt} + \text{inflow})}_{\text{water sources}} - \underbrace{(\text{interception} + \text{outflow} + \text{AET})}_{\text{water sinks}} - \Delta\ \text{soil moisture} = \text{INFILTRATION} \qquad (1)$$

Spatially gridded datasets required for SWB simulations include land cover, overland flow direction, hydrologic soil group, available soil-water capacity, daily precipitation, daily maximum temperature, and daily minimum temperature. Tabular information required by SWB include runoff curve numbers, vegetation rooting depths, interception values, and maximum daily recharge values for each combination of hydrologic soil group and land-cover type. Inflow to a cell is surface flow from adjacent cells, calculated using the Natural Resources Conservation Service (NRCS) curve number rainfall-runoff relation [6]. The direction of runoff from cell to cell is determined using a flow-direction grid derived from a digital-elevation model (DEM). Interception is a user-specified amount of precipitation that is trapped and used by vegetation. Outflow from a cell is calculated in the same manner as inflow to the cell. There are several methods available for estimating potential evapotranspiration (PET) in the SWB model, from which actual evapotranspiration (AET) is calculated. For the Grand Canyon-area simulations, the Hargreaves-Samani [7] method is used as it produces spatially variable estimates of potential ET (PET) from spatially varying minimum and maximum air temperature data for each daily time step:

$$\text{PET} = 0.0135 \times \text{RS} \times (\text{T}+17.8) \quad \text{with RS} = \text{KRS} \times \text{RA} \times \text{TD}^{0.5} \qquad (2)$$

where PET is potential ET, RS is incoming solar radiation, T is mean air temperature in °C, KRS is a calibration coefficient, RA is extraterrestrial radiation, and TD is the measured air temperature range [7]. Extraterrestrial radiation is estimated as a function of the day of year and latitude [8]. The computation of soil moisture in equation 1 requires several intermediary values. First, PET is subtracted from precipitation (P) for all grid cells. If P – PET is negative (i.e., if P < PET), then there is a potential deficiency of water. Accumulated Potential Water Loss (APWL) is computed as the running sum of daily P – PET values during times when P < PET. Soil moisture is estimated using the current AWPL value in the Thornthwaite-Mather relation that describes the nonlinear relation between soil moisture and APWL. Actual ET (AET) is then equal to only the amount of water that can be extracted from the soil. If P – PET is positive (i.e., if P > PET), a potential surplus of water exists and AET is equal to PET. Soil moisture is calculated by adding P – PET directly to the previous day's soil-moisture value. If the new soil moisture value is less than the maximum water-holding capacity of the soil (calculated as the product of the available soil water capacity and the root-zone depth), then the Thornthwaite-Mather relation is used to back-calculate a reduced APWL. If the new soil moisture value is greater than the maximum water-holding capacity of the soil, then soil moisture is capped at the maximum water-holding capacity, excess soil-moisture becomes recharge, and AWPL is set to zero.

All spatially gridded input datasets were resampled to the same cell size and geographic coordinate system as the 1/8th degree Coupled Model Intercomparison Project 5 (CMIP5) climate data. Detailed descriptions of the sources, manipulation, and resampling of SWB model inputs for upper Colorado River basin recharge simulations, which use the same data sources and processes as Grand Canyon-area simulations, are provided in *Tillman* [9].

Climate changes are expressed in SWB simulated recharge results (equation 1) through the computation of AET (mean temperature) and through precipitation input. The SWB model does not include changes in land use over time or simulate changes in stomatal conductance or leaf area in a CO_2 enriched atmosphere [10, 11]. Only direct effects of climate change are evaluated in SWB recharge results.

While the SWB model has been shown to provide reasonable basin-scale estimates of groundwater recharge, SWB limitations and assumptions warrant consideration when evaluating simulation results [3]. The daily time step of the SWB model allows short-term surpluses of water to become recharge, but also necessitates that overland-flow routing of runoff either

infiltrate in cells downslope or be routed out of the model domain on the same day in which it originated. Depth to the top of the aquifer surface also is not considered in SWB, and there may be substantial time of travel through the unsaturated zone. Use of the NRCS curve number method to estimate runoff in SWB introduces limitations, including that the method was developed to evaluate floods and was not designed to simulate daily flows of ordinary magnitude, and studies that show that the curve number is not constant but may vary from event to event [3]. Finally, there are numerous methods for estimating groundwater discharge by evapotranspiration, each with its own benefits, limitations, uncertainties, and data requirements. This study uses the Hargreaves-Samani [7] method for PET, in which climate changes are reflected only in the daily air temperature range. More complex ET relations require additional data including relative humidity, wind speed, and percentage of actual to possible daily sunshine hours, among others [3].

References

1. Vose, R. S. *et al.* Gridded 5km GHCN-Daily Temperature and Precipitation Dataset (nCLIMGRID). *NOAA National Centers for Environmental Information.* https://doi.org/10.7289/V5SX6B56 (2014).
2. Vose, R. S. *et al.* Improved historical temperature and precipitation time series for U.S. climate divisions. *J. of Appl. Meteorol. Clim.* **53**, 1232–1251. https://doi.org/10.1175/JAMC-D-13-0248.1 (2014).
3. Westenbroek, S. M., Engott, J. A., Kelson, V. A., & Hunt, R. J. SWB Version 2.0—A soil-water-balance code for estimating net infiltration and other water-budget components. *US Geol. Surv. Tech. Methods*, book 6, chap. A59, 118 p. https://doi.org/10.3133/tm6A59 (2018).
4. Thornthwaite, C. W. An approach toward a rational classification of climate. *Geogr. Rev.* **38**(1), p. 55–94 (1948).
5. Thornthwaite, C. W. & Mather, J. R. Instructions and tables for computing potential evapotranspiration and the water balance. Centerton, N.J., Laboratory of Climatology, *Publications in Climatology*. **10**(3), 185–311 (1957).
6. U.S. Department of Agriculture. Chapter 9, Hydrologic Soil-Cover Complexes *in* Part 630 Hydrology, National Engineering Handbook, 210-VI-NEH. 20 p., Available at https://directives.sc.egov.usda.gov/OpenNonWebContent.aspx?content=17758.wba (2004).

7. Hargreaves, G. H. & Samani, Z. A. Reference crop evapotranspiration from temperature. *Appl. Eng. Agric.* **1**(2), 96–99. https://doi.org/10.13031/2013.26773 (1985).
8. Allen, R. G., Pereira, L. S., Raes, D. & Smith, M. Crop evapotranspiration—Guidelines for computing crop water requirements. *FAO Irrigation and Drainage Paper.* **56**, 333 p. http://www.fao.org/docrep/x0490e/x0490e00.htm (1998 with errata 2006).
9. Tillman, F. D. Documentation of input datasets for the soil-water balance groundwater recharge model for the Upper Colorado River Basin: *US Geol. Surv. Open-File Rep.* 2015–1160, 17 p. https://doi.org/10.3133/ofr20151160 (2015).
10. Eckhardt, K. & Ulbrich, U. Potential impacts of climate change on groundwater recharge and streamflow in a central European low mountain range. *J. Hydrol.* **284**, 244–252. DOI:10.1016/j.jhydrol.2003.08.005 (2003).
11. Holman, I. P., Allen, D. M., Cuthbert, M. O. & Goderniaux, P. Towards best practice for assessing the impacts of climate change on groundwater. *Hydrogeol. J.* **20**, 1–4. https://doi.org/10.1007/s10040-011-0805-3 (2012).
